# Structure of the pork value chain in Northern Uganda: implications for *Taenia solium* control interventions

**DOI:** 10.3389/fvets.2023.1177526

**Published:** 2023-05-22

**Authors:** Nicholas Ngwili, Salaviriuse Ahimbisibwe, Derrick Noah Sentamu, Lian F. Thomas, Emily Ouma

**Affiliations:** ^1^International Livestock Research Institute, Nairobi, Kenya; ^2^International Livestock Research Institute, c/o Alliance-Bioversity International-CIAT, Kampala, Uganda; ^3^Institute of Infection Veterinary and Ecological Sciences, University of Liverpool, Neston, Wirral, United Kingdom

**Keywords:** *Taenia solium*, cysticercosis, pork value chain, Northern Uganda, one health

## Abstract

**Introduction:**

This study characterizes the pork value chain in Agago, Kitgum, Lamwo, and Pader districts of Northern Uganda and analyzes its implications on the transmission and control of *Taenia solium* infections.

**Methodology:**

Data were collected through focus group discussions (FGDs) with farmers and pig and pork traders, key informant interviews (KIIs) with local government officials and consumers, and district-level multi-stakeholder mini workshops organized in the study area. The value chain actors identified include input and services providers, pig farmers, live pig traders, pork traders, and consumers.

**Results:**

Most of the pig production, marketing and consumption was found to occur through informal channels. Pig production in this area is dominated by smallholder extensive systems, with most producers keeping less than 10 pigs. The production segment of the pig value chain is characterized by low use of inputs and services such as veterinary extension, drugs and improved feeds. Pigs scavenge for food under free-range systems and are at risk of parasitic infections such as the zoonotic helminth *Taenia solium*. This risk is exacerbated by the inherent contextual aspects of the study sites including low latrine coverage, open defecation, and high poverty. In addition, some respondents viewed pigs as “sanitation policemen” where they leave them to roam around and eat dirt including feaces thereby cleaning the environment. *T. solium* was recognized as an important pig health constraint in this value chain alongside African swine fever (ASF). Unlike ASF that was associated with the pig mortalities, the cysts were associated with rejection of pigs by traders at purchase, condemnation of pig carcasses by meat inspectors and rejection of raw pork by consumers at points of sale.

**Discussion:**

Poor organization of the value chain and lack veterinary extension and meat inspection services results in some pigs infected with *T. solium* entering the food chain, exposing consumers to infection with the parasite. To reduce the pig production losses and public health impact from *T. solium* infections, there is need for control and prevention interventions targeting specific nodes of the value chain where the risk of transmission is highest.

## Introduction

Pig production is one of the fastest growing livestock sectors worldwide and this trend is expected to continue over the coming years (1). Uganda, an East African nation, is experiencing a rise in demand for pork in both domestic and regional markets. Neighboring countries such as South Sudan, the Democratic Republic of Congo (DRC) and Rwanda imported 167 tonnes of pork in 2011 from Uganda (2). Uganda has the highest *per capita* consumption of pork in Eastern Africa, at 3.4 kg/person/year in 2007 (3). In response to the increase in demand for pork, Uganda’s pig production has been growing rapidly over the last decade. Pig population increased from 3.18 million in 2008 to 4.04 million in 2016 (3). The sector supports the livelihoods of over 1.1 million pig farmers and several other value chain actors such as pig traders, veterinary drug shop operators, pig transporters, and feed stockists (4).

A value chain is defined as the set of activities which are undertaken to bring a product or service from production, through various stages including transformation into a sellable product, its delivery to final consumers, and final disposal after use (5). Therefore, the pig value chain describes all the activities from production, trade, slaughter, processing, consumption of pigs and pig products including pork, and disposal of waste. In the value chain the different actors and their roles can be identified and described through a process called value chain mapping (6). The different segments of the value chain implementing specific functions are referred to as nodes, and are points where particular value chain actors operate and exert influence (5).

Pig production in Uganda is dominated by smallholder semi-intensive and extensive production systems such as tethering, free-range, partial confinement, or a mix of these (2, 7). The raising of pigs under extensive system coupled with poor sanitary practices by the pig keeping communities predisposes pigs to infections including the zoonotic parasites such as *Taenia solium*, *Trichinella spiralis* and *Toxoplasma gondii*, as well as key production and trade limiting diseases such as African Swine fever (ASF) (8, 9).

*Taenia solium* (pork tapeworm) is a neglected zoonotic parasite that causes serious public health and socio-economic burden in developing countries (10). *T. solium* infection results in three different illnesses: Porcine cysticercosis (PCC) in pigs, and Taeniasis and Neurocysticercosis (NCC) in humans (11). Neurocysticercosis is the leading cause of preventable adult-acquired epilepsy in sub-Saharan states, including Uganda (9). Being a zoonotic disease, *T. solium* is one of those diseases whose sustainable control measures requires the adoption of the One Health approach (12).

At the global level, *T. solium* accounts for over 2.8 million disability-adjusted life-years (DALYs), with the highest burden shouldered by the already impoverished and neglected communities in different regions of the world (13). For Uganda, it has been estimated that NCC results in more than 170,000 DALYs and 75 million USD in health burden and economic losses, respectively (14). Interventions to break the parasite transmission cycle can be grouped into mass human antiparasitic therapy or treatment of individuals patients, antiparasitic treatment of pigs, improved meat inspection, human health education on proper personal and household hygiene, proper cooking of pork and good pig husbandry (15, 16). Although a vaccine for *Taenia solium* infections in pigs exists, and would be an important control tool, it has not yet been adopted in Uganda and is not commercially available (17).

For porcine cysticercosis, the prevalence and attempts to control the infections may be influenced by the inherent challenges at different nodes of the pork value chains in Uganda. The challenges in the value chains include lack of quality pig feeds (well formulated and nutritious pig feed), limited access to veterinary and breeding services, limited enforcement of meat inspection, limited investment in pig housing, and limited farmer knowledge on proper pig husbandry and biosecurity practices (4, 18–21). Most of the raw pork (not cooked) sold in Uganda’s local markets is not inspected, and pigs are slaughtered in unhygienic conditions in slaughter areas with poor waste disposal systems, thereby posing a high risk of zoonotic infections (4). At the production node, the uptake of best practices and inputs and services by pig producers is limited leading to adoption of semi-intensive and extensive production systems characterized by poor feeding and lack of housing which are known risk factors for PCC (4, 22). The predominance of informal pork value chain processes characterized by a lack of organized trading channels, backyard slaughter points, and markets which are unresponsive to consumer preferences on pork products coupled with poor enforcement of meat inspection regulations, predisposes consumers to taeniasis, which perpetuates the *T. solium* life cycle (4, 8, 23, 24). The outlined challenges combined with poor sanitation and poverty predispose pigs and humans to infections with *Taenia solium* cysticercosis and taeniasis, respectively.

The control of *T. solium* calls for understanding of contextual issues at the specific target areas and multisectoral approach involving all relevant stakeholders (12, 25). Value chain analysis supports the identification of the constraints in the different nodes of the chain as well as helps in the design the potential interventions. Several studies have attempted to map the pig value chains in the East Africa region including Kenya, Uganda and Rwanda (4, 6, 26). The three studies mapped the pig value chain structure, identify the governance and operational challenges, none of them focused on a specific zoonotic parasite. In the current study, the approach described in Baltenweck et al. (27) which entails collecting data using Focus group discussions (FGD) and Key informant interviews (KIIs) with different actors along a predetermined conceptualization of the pig value chain is used. The aim of the study was to describe the structure of the pork value chain in Northern Uganda to identify potential intervention points to control *Taenia solium* infections at the different nodes.

## Materials and methods

### Research approval

Research ethical review and approval for this study were obtained from the International Livestock Research Institute (ILRI) Research Ethical committee (Ref. ILRI-IREC2022-09) and Vector Control division research ethics committee under the Ministry of Health, Uganda (Ref. VCDREC156). Additionally, research clearance was granted by the Uganda Council of Science and Technology (Permit No. UNCST: SS1296ES). Informed consent was sought from all research participants, and they signed the informed consent forms with the help of a local language translator.

### Study area

**Figure 1:**
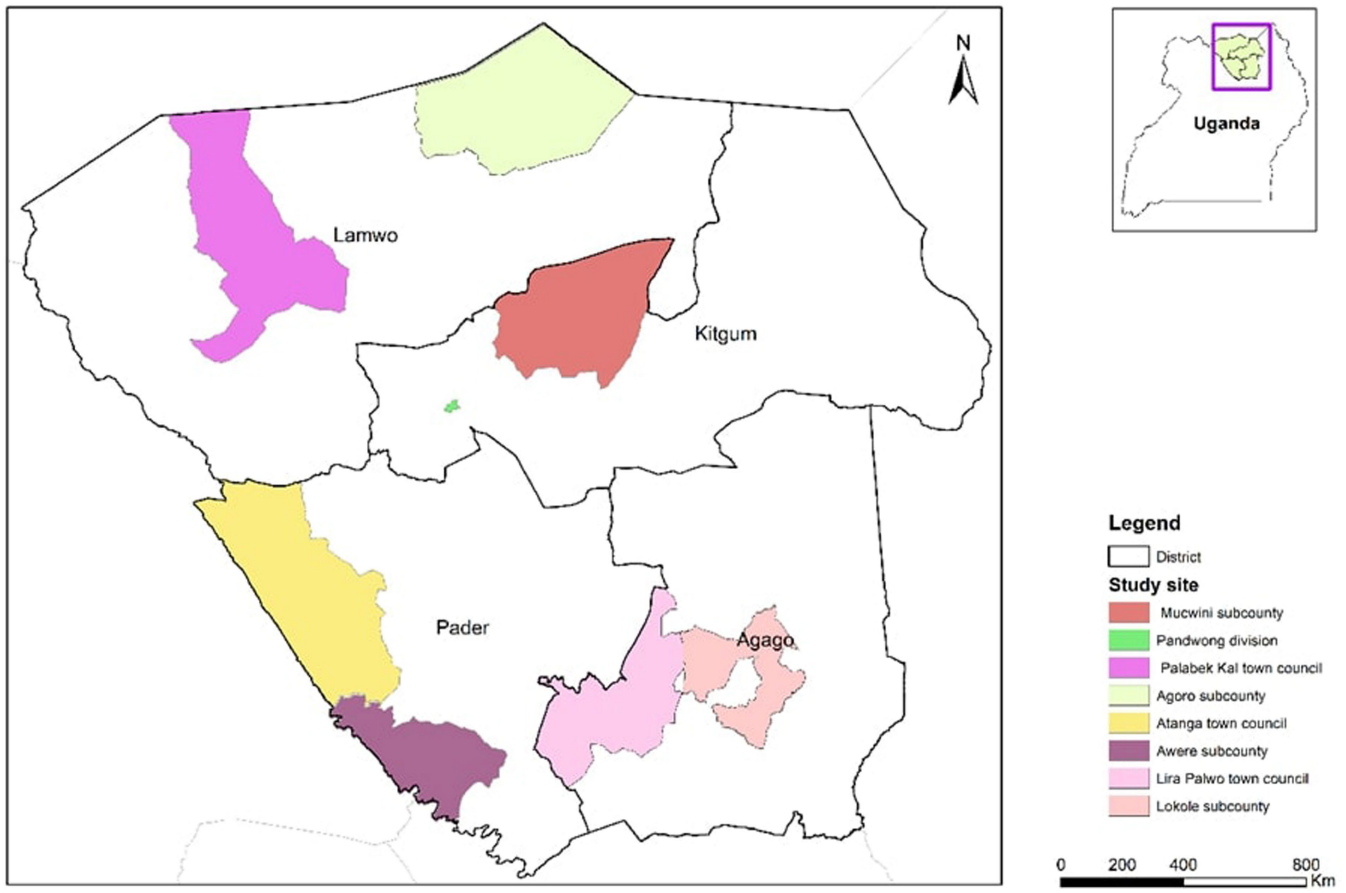
Map of the study site.

**Figure 2:**
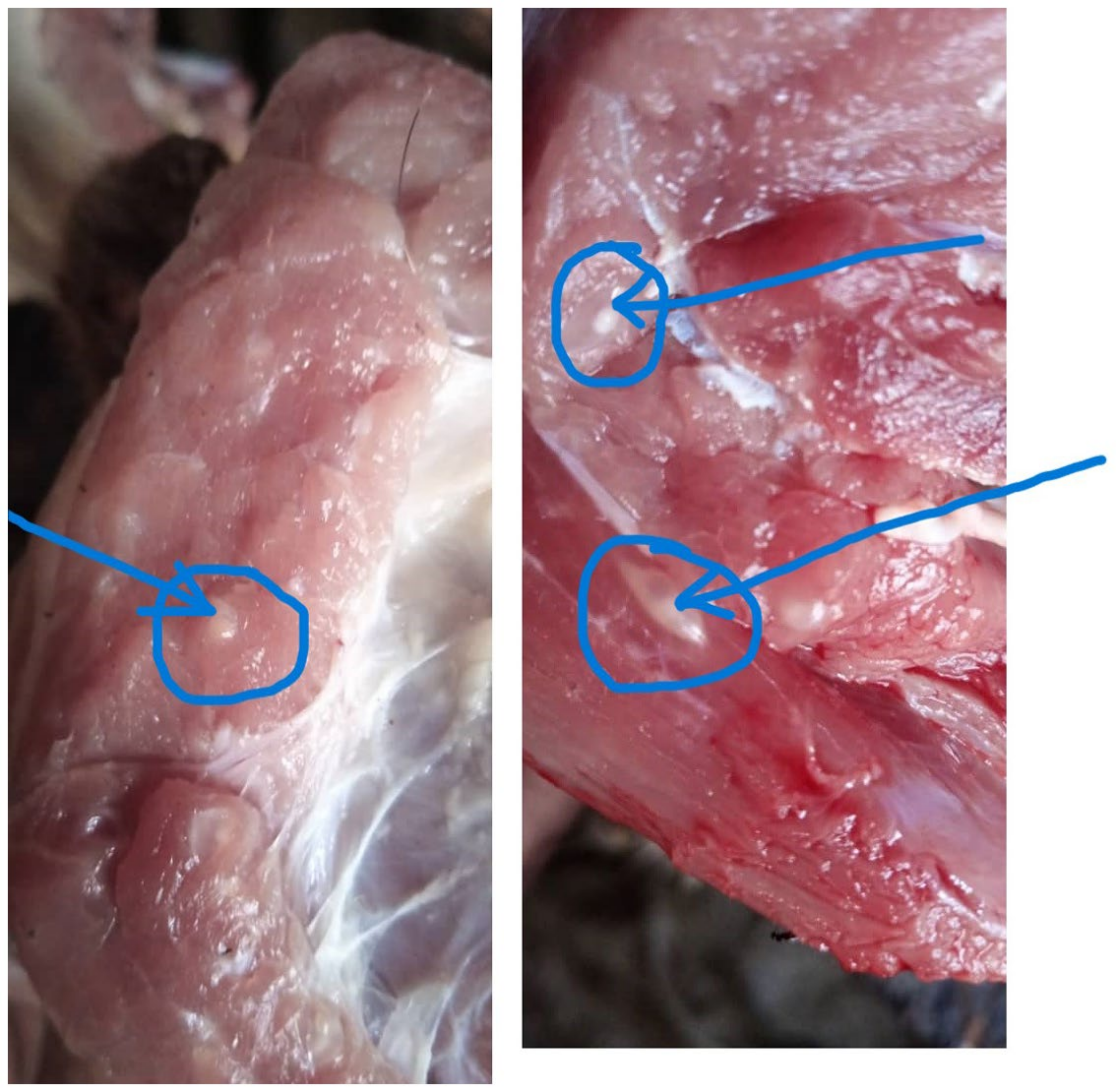
Pork meat infested with *T. solium* cysts found by a meat inspector in Lamwo district (Photo courtesy of Kidega Samuel).

A cross-sectional qualitative study was conducted in four districts of Northern Uganda in May and June 2022. The districts included Kitgum, Lamwo, Pader and Agago (Figure 1). In each district 2 sub counties were selected, one rural and the other peri urban. The districts were initially selected based on the congruence of three risk factors namely, sanitation, pig population density and poverty levels as described in Ngwili et al. (28). Additionally, anecdotal reports from meat inspectors trained under the ILRI’s Boosting Uganda’s Investments in Livestock Development (BUILD) project and other meat inspectors in the study area indicated a considerable proportion of the pork tapeworm cases in slaughtered pigs detected during routine meat inspection, flagging these districts as potential hotspots (Figure 2). Finally, a scoping/scouting visit was conducted in several districts in the region and discussions with the relevant stakeholders confirmed the region as potentially having high prevalence of the *Taenia solium* infections in both pigs and humans. Region here refers to the 5 districts visited during the scoping visit to scout for potential study sites. Scoping visit is a fact-finding mission used to gather more information to aid decision making, in this case the decision was on which districts to include as potential study sites.

### Selection of participants

Data were collected through focus group discussions (FGD), mini workshops, and key informant interviews (KIIs). This study targeted actors at the different pork value chain nodes based on the methodology described in Baltenweck et al. (27). Actors as used in this context refer to people who play key roles in the value chain. The FGD and KII guides were based on the toolkit suggested by Baltenweck et al. (27) for value chain analysis but modified to focus on issues on the pig value chain that are relevant to the *T. solium* infections and control.

#### Focus group discussions

The participants for the pig producer node were identified with the help of the subcounty extension workers and were selected from across each of the selected sub-county and from different villages representing the various pig production systems prevalent in the area. The FGDs for pig producers were divided according to gender and sub-county with each FGD having a maximum of 12 participants as recommended by different studies (29) (Table 1). For the pig traders, the participants were identified by the district veterinary officer and were drawn from across the district due to the small number of traders in each sub county. A total of four (4) trader FGDs were conducted.

**Table 1:** Participant information.

Stakeholder category	Tool	Total number of participants	Male participants	Female participants
Producers	FGD	173	88	85
Pig traders	FGD	45	43	2
Veterinary and extension officials	KII	12	11	1
Community development officers	KII	4	3	1
Consumers	KII	15	11	4
Stakeholders from different nodes*	Mini workshop guide	78	53	25

KII-Key Informant Interviews, FGD-Focus Group Discussion; nodes*- production, inputs and services, pig and pork trade, pork consumption.

#### Key informant interviews

Select public officials relevant to the pork value chain were identified based on the initial opinion of the District Veterinary Officers’ (DVOs’) and previous pork value chain studies in Uganda (4, 30) to be interviewed as key informant interviews. These were conducted with pork consumers (15), veterinary extension officials (12) representing the district veterinary officer and animal health assistants in charge of sub-counties and community development officers (4). The consumers were identified and interviewed in various settings (e.g., roadside pork roasters, improved pork joints, and pork butcheries).

#### Mini workshop

For each district, a multistakeholder mini workshop composed of about 20 stakeholders from the various nodes of the pork value chain was conducted in a central location within the districts. The mini workshop aimed to bring together stakeholders from various nodes to reach a consensus on any issues that may have been contentious in the FGDs. Those invited to the mini workshops were selected as representatives from the FGD and KII participants. The participants that were invited for the mini workshop consisted of 6 farmers, 4 extension workers/meat inspectors, 4 Traders/transporters, 2 commercial officers, 1 public health official, and 2 Agrovet dealers and the district veterinary officer.

### Data collection

The FGD and KII guides were developed, reviewed by one of the senior authors who is an expert in pork value chain analysis (EO) and pretested in one of the sub counties outside the targeted study sites. The guides for the FGDs and KIIs except the consumers’ KIIs guide started with an activity to map the value chain using cards followed by a discussion on the actors at the different nodes and their roles. The key nodes in the value chain were identified *a priori* to align with those in Baltenweck et al. (27). This was followed by a discussion of the key constraints along the value chain and the opportunities which exist in the value chain. Special emphasis was made to bring out aspects related to *Taenia solium* infections and control.

The consumer KII guide focused on pork consumption, their preferences and food safety issues. The FGDs were conducted by two locally recruited facilitators in the local Luo-Acholi dialect. The KIIs were conducted by one of the research team members (SD). All the FGDs and KIIs were audio-recorded, and pictures of other important observations were also taken. Data was recorded as audio files then transcribed to text files.

### Data management and analysis

The audio-recordings were translated from Acholi to English and transcribed verbatim into Microsoft word. The data was imported and analyzed in NVivo® qualitative analytics software^1^ to summarize related themes and describe the value chain map in detail. The process used in mapping the value chain utilized the value chain framework and tools drawn from Baltenweck et al. (27) that guides the discussions on the flow of the pig/pig products until consumption, the inputs and services used in piggery, suppliers of inputs and services, and the transformation of the pig and pig products along the chain. A coding frame was then developed by the two lead authors and the data characterizing each node of the value chain coded deductively to explain the structure of the pig value chain and the underlying practices. The results were then used to tease out the risks to *T. solium* infections along the different value chain nodes and the implications on the parasite control. The value chain maps were developed using Cmaps® software.^2^

## Results

Pig value chain analyzes and mapping having been conducted in different settings in the East Africa region; Ouma et al. (4) in Uganda, Shyaka et al. (6) in Rwanda, and Murungi et al. (26) in Kenya. However, to the best of our knowledge this is the first study to describe the pig value chain in relation to *Taenia solium* infections and control. The results section first provides an overview of the pork value chain, based upon the data collected in this study. We then highlight specific opportunities and constraints related to *T. solium* infection and control at each node of the value chain. A conceptual framework showing where the potential risk for *Taenia solium* infections exist along the different nodes of the value chain is shown and discussed pointing to potential areas of interventions and the actors to be targeted participant information.

A total of 16 gender disaggregated (divided as per the gender of the respondent) focus group discussions were conducted with pig producers (eight female participant FGDs and eight male participant FGDs), aged between 18 and 69 years (Table 1). Most of the participants had attained at least primary level of education. There were fewer women (2/16) posted by public veterinary and commercial services in lower local governments as compared to men (14/16) in the areas that were selected for this study. Fifteen consumers were also interviewed across the four districts.

### Overview of the pork value chain in the selected districts

**Figure 3:**
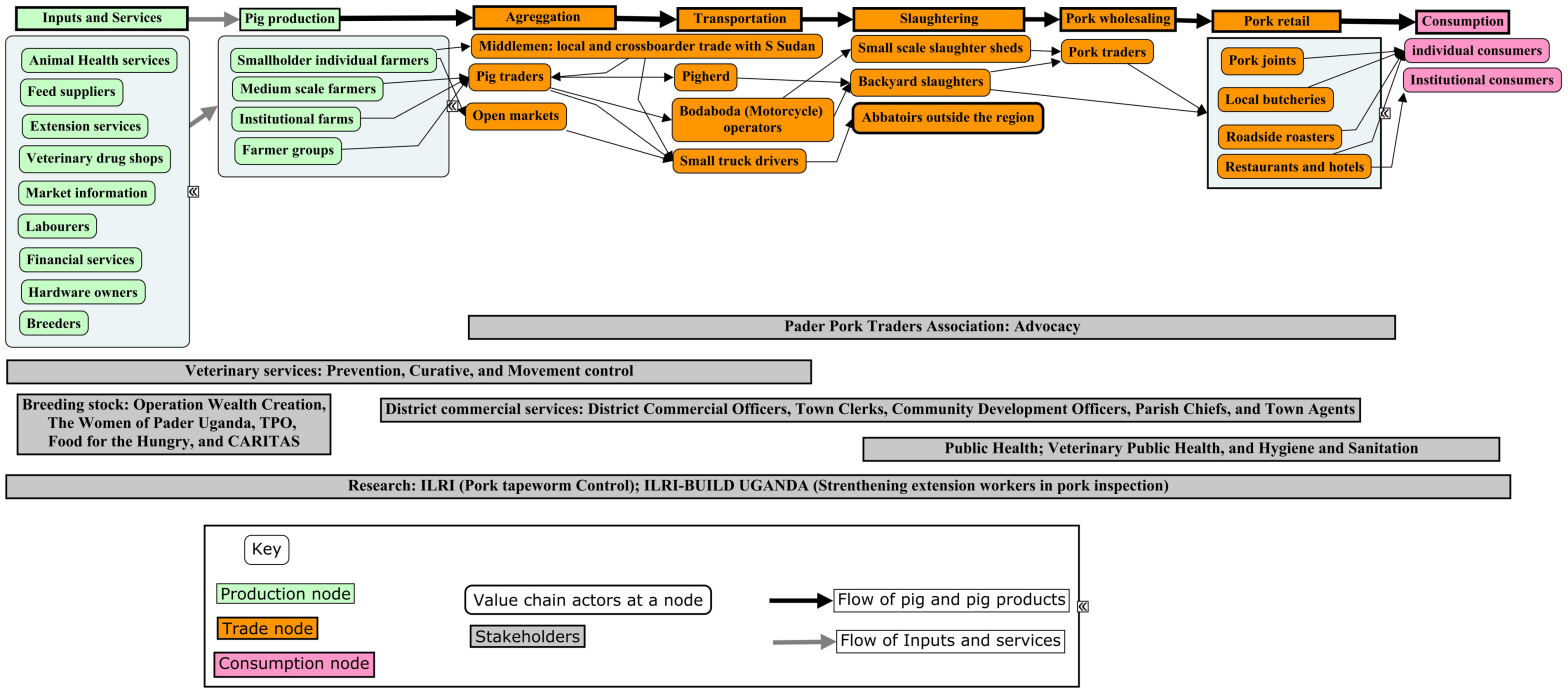
Pork value chain map for Agago, Kitgum, Lamwo and Pader districts.

The value chain was perceived by stakeholders as being poorly organized with many independent actors operating in an uncoordinated way at various levels and scope, from production through trade to consumption. The pork value chain actors identified by the stakeholders were input and services providers, animal health and extension workers, producers, aggregators, transporters, pig traders, pork traders, pork joint operators, public health officials, commercial services providers, and consumers as shown in the aggregated pork value chain in the four districts (Figure 3). For some of the nodes, several actors operate there depending on the roles they play. For example, under inputs and services: feed/breeding/health, production, pig producers were involved, at live trade node–aggregators, transporters, traders; at slaughter–pig traders and meat inspectors are involved, at wholesale–pork traders are involved, at pork retail–pork joint operators were involved and finally at consumption consumers were involved. Pork joints are roadside eateries which sale ready to eat pork mainly roasted and sometimes served with raw vegetables.

**Figure 4:**
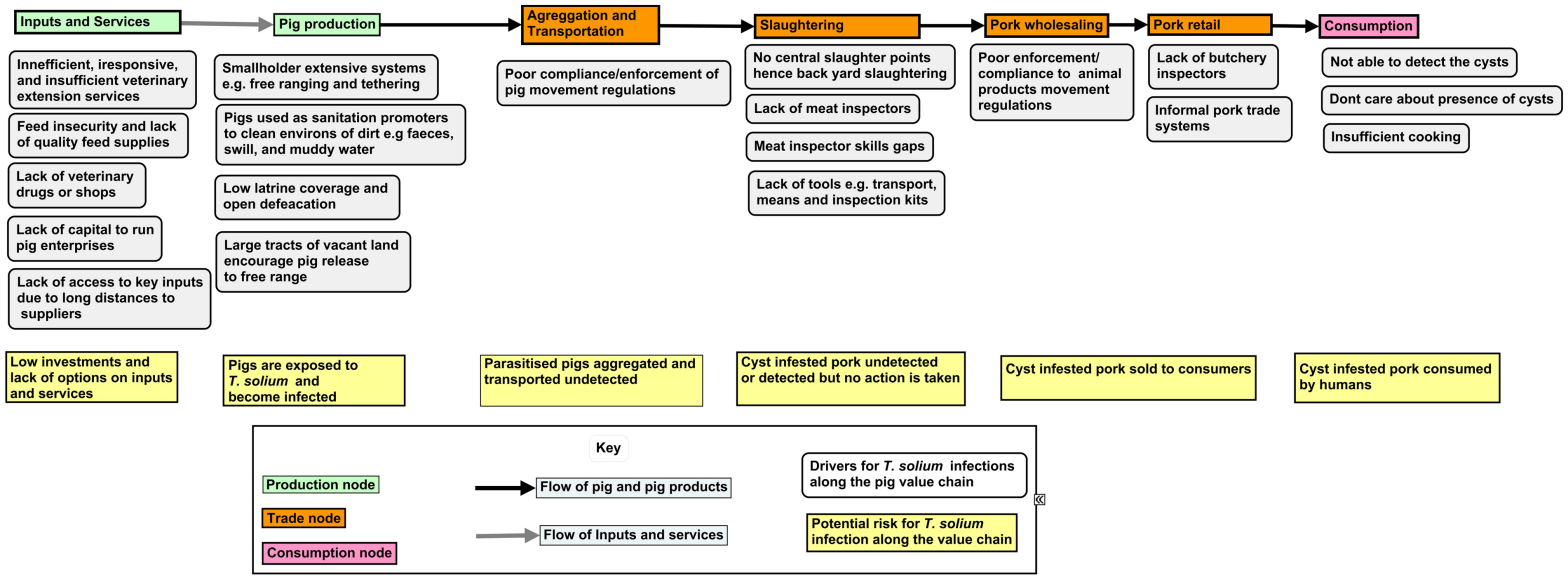
Conceptualizing *T. solium* infections along the nodes of the pork value chain in Northern Uganda.

The stakeholders also identified several challenges along the different nodes of the value chain with diseases being an important constraint. In this study we focus on porcine cysticercosis in pigs caused by the intermediate stage of the zoonotic parasite *T. solium.* We report the drivers and the risks of infections along the different nodes in the pork value chain in four districts in northern Uganda as identified by the different stakeholders interviewed. The value chain nodes at which the drivers and risks for infection exist, and where interventions could be targeted are outlined (Figure 4).

### Inputs and services

#### Breeding

The sources of stock for breeding were fellow farmers, government programs such as Operation Wealth Creation (OWC), institutional farms like the Kitgum prison farm, and goodwill politicians. There were diverse options for servicing of the sows with the commonest being free ranging that allowed pigs to mix up and mate with other roaming boars in the village. It was also reported that the government at times provided improved boars, with better genetic traits and often exotic, which are left roaming around enabling cross breeding with the local breeds. Some of the producers believed that the pork tapeworm was transmitted from improved boars to sows at service which is not correct with regards to PCC transmission.

#### Feeds and feeding

Various feeding strategies were used by the producers, but the key characteristic was the seasonality of the feeds, mostly crop residues. In the wet seasons when the pigs must be tethered or confined to avoid destruction of crop fields, the feed sources were maize bran, rice bran, and sunflower seed cake from grinding mills, brewers waste, kitchen waste, plant residues especially cassava peels and leaves, and free-growing shrubs and vegetables. Farmers also reported going to open markets to buy ingredients such as silver fish waste.

In the dry seasons, most producers preferred free range. Feed insecurity was commonly reported by most stakeholders as a constraint that leads to low grade pigs and is the key driver of free ranging production systems which predisposes pigs to diseases. Most participants in the different nodes also observed that there was a lack of actors in the feed supplies node, making it hard to find a reliable source of quality commercial feeds. Most producers in the rural settings cited that they lacked finances to buy commercial pig feeds. For farmers who rely on crop residues as feed for pigs, the seasonal availability of feeds is an important constraint.

Some participants believed that allowing pigs to free range on dirty materials like swill, feces, and stagnant muddy water made them appear healthy. It was reported by some participants that in some of the communities, pigs were referred to as ‘sanitation policemen’ or ‘hygiene promoters’ as producers release them to ‘clean’ the environs of dirty things such as feces, and swill.

*“Agago as district is still having high rate of open defecation like if you go to some other villages, you will hardly see pit latrine so that means they still use open defecation and to some extent, such areas are found to be having many pigs so, some farmers always refer to them as hygiene promoters or sanitation policemen because sometimes the waste are put there and it’s the [job] pigs to pick,”* District Veterinary Officer KII.

*“For us in the village also, we might stay for even two months without giving our pigs feeds, we leave them [free range] to look for their own feeds,”* Participant, Kitgum district mini workshop.

#### Animal health services

Veterinary services were reported to be provided by public veterinarians and para-veterinarians, private para-veterinarians, farmers, and in some areas, NGOs (Non-Governmental Organizations). The reported functions of veterinary services were farmer advisory services, pig health monitoring and treatment, and vaccination. Some stakeholders also mentioned that veterinary services were important in inspection, regulation, and the control of the movement of pigs and pork.

*“When my pigs are sick, I call a veterinary doctor who comes and assess them and give them the necessary treatment,”* P10, female producer FGD, Agago District.

*“When I plan to sell my pig, I call for the veterinary doctor to check the health of my pigs more especially Taenia solium, having cysts under the tongues of the pig, before looking for the buyer,”* P5, male producer FGD, Kitgum district.

Most of the producers noted that they do not have access to sufficient veterinary services such as treatment, extension, and vaccination. Even the available veterinary services were reported to not respond when notified of disease outbreaks.

*“And the other thing is advice from our extension service provider is very hard to get advice from them, to be honest the extension service providers we have here don’t help us with advisory services,”* said P7, male producer FGD, Kitgum district.

*“Like in this district, veterinary staffs are only 8, including the DVO and yet we have 19 sub counties and town council,*” District production officer, KII.

Additionally, veterinary drug shops were mentioned as important sources of farm equipment, feeds and feed ingredients, and veterinary drugs such as dewormers, acaricides, disinfectants, and antibiotics. Most participants mentioned that they were referred by animal health workers or sought assistance from the veterinary drug shops when their pigs needed clinical attention. Farm equipment mentioned included ropes for tying the pigs, rakes, hoes, wheelbarrows, water tanks, and gum boots. However, it was also reported that where drug shops are far, they buy these drugs and inputs from open markets where drugs are displayed on market days. Some rural areas reported that they lacked veterinary drug shops and had to move long distances to larger urban centers to find one.

“*Even on market days you buy the drugs from there [open market], like on auction days drugs are displayed there*,” Participant, Pader district Mini workshop.

### Production

**Figure 5:**
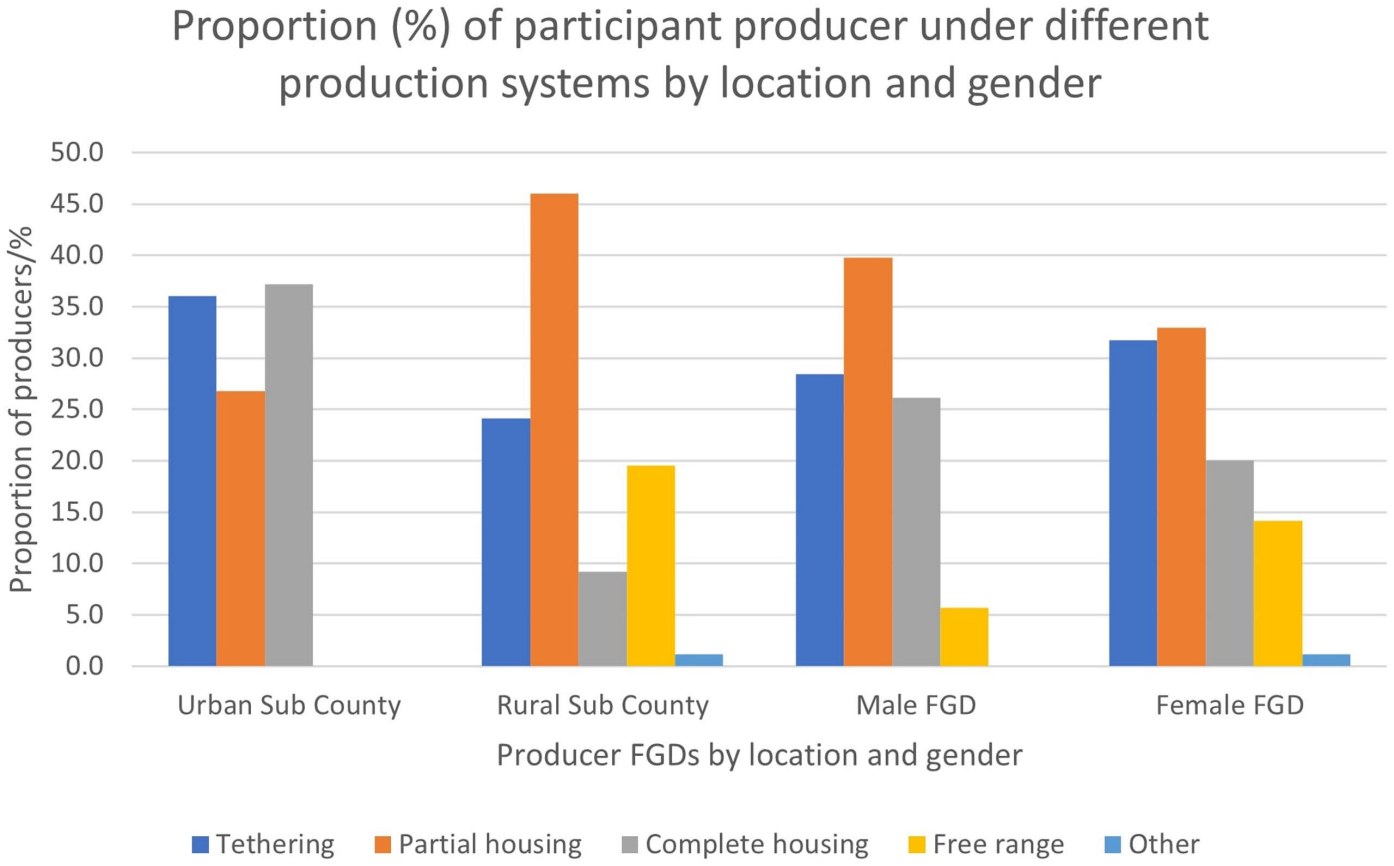
The proportion of respondent producers under different production systems.

Pig production is dominated by subsistence smallholder farmers (80–90%) while the remainder are medium (7–20%) or large-scale producers (0–3%) (Table 2). The participants in the stakeholder mini workshop categorized the sizes of the farms as follows: smallholder (<10 pigs); medium scale (10–30 pigs); and large scale (>30 pigs). Rural farmers practiced extensive production systems characterized by seasonal free ranging and tethering, while urban and peri urban producers were commonly semi-intensive with pigs being confined (Figure 5). There were also a few institutional farms managed by schools, churches, and Uganda prisons which were reported to be intensive, rearing improved breeds.

**Table 2:** Proportions of holding sizes and production systems.

District	Herd size/%			Production system/%		
	Small	Medium	Large	Extensive	Semi intensive	Intensive
Agago	75	20	5	61	35	4
Kitgum	90	9	1	35	65	–
Lamwo	80	20	0	80	15	5
Pader	50	40	10	80	20	–

Production methods were also determined by the seasons of the year, dry or wet which drive the availability of natural feeds. Most stakeholders understood that free ranging pigs in dry season was associated with outbreaks of ASF and, to a few, the pork tapeworm. Producers also reported that they lacked knowledge of disease control and prevention, identifying quality inputs, feeding, and construction of pigsties.

“*For us here we have two seasons that is; period for cultivation/planting we tether and put them in the house and in dry season when there is not much crop in the garden then we leave them on free range systems,*” Participant, Agago District Mini workshop participant.

*“Another problem here is that most of us keep our pig under free range system, and that why our pigs are infected with diseases because they mix with the sick pigs,”* P5, male producer FGD, Kitgum district.

Housing was reported as an essential element of pig production, but most producers had no dedicated pig housing, instead they reported that often pigs are tied under broad leaved trees near the homesteads rather than being housed. Housing was mostly necessary to stop pigs from roaming around the village, not to destroy the crop field but are deemed less necessary after harvesting and during the dry season. Partial housing was a common practice in both rural and urban areas and involves releasing the pigs to free range at certain times of the day and then housing them again. Hardware shops were mentioned as important sources of materials used in pigsty construction by the farmers who have pigsties, and most farmers cited lack of money to buy construction inputs as the reason they could not afford permanent pigsties.

*“I do agree on the ideas of P10[another participant] because the system that we are using for raising our pigs is not safe, for example tethering and free-range system, simply because we can’t afford to construct a good house for our pig, hence diseases come and destroy our pigs,”* P1, male producer FGD, Lamwo district.

Various diseases were identified as being constraints to production because of resulting deaths, reduced productivity, and condemnation of pigs and pork at sale. Stakeholders mentioned that the most prevalent diseases in the area include ASF, *T. solium* (referred to by stakeholders as “pork tapeworm,” “condemned,” “fallen,” local terms such as “opoto” or “owak” or with a description of the cysts under the tongue and in the meat), external parasites, intestinal worms, and other unidentified diseases described by the participants through symptoms like coughing, diarrhea, and hair loss. While most participants identified ASF as the leading cause of production losses at the farm through death of pigs and loss of income, the pork tapeworm was mostly associated with losses resulting from rejection of infected live pigs by buyers at farm level, condemnation of pork at slaughter and sales points, and refusal by consumers to buy pork infected with cysts.

*“To me, the problem which affects sales is the disease [pork tapeworm] which attacks our pigs, when buyers come to buy our pigs, they check under the tongue, so when they find cysts, they do not buy such pigs”* p8, female producer FGD, Agago District.

*“I slaughtered mine in April this year [2022] and I found a lot of cysts in it, even the veterinary doctor did not inspect, we just threw it away because the cysts were too many and could not be eaten,”* P8, female producer FGD, Pader district.

*“Some of our pigs are affected with worms commonly known as ‘opoto/condemned’ [pork tapeworm] that either can be found under their tongue or in their muscles, and it is the one affecting our sale mostly because people will physically see the worm in the meat then peoples reject our meat. We commonly experience this [pork tapeworm infection] when our pigs are on free-range system in the dry season,”* P1, male producer FGD, Kitgum district.

### Live pig trade

Live pig trade encompasses the collection, aggregation, and transportation of live pigs from individual producers to open air pig markets and slaughter points within or outside the district. Aggregation was reported to be conducted by pig traders, producers, and middlemen. The middlemen were categorized as referral agents or commission agents and were mostly associated with large volume traders from neighboring cities such as Gulu, Lira, Kampala, and neighboring DRC and South Sudan. Once the pigs have been aggregated, they were transported to centers of slaughtering. The means of transport depended on the distance to the destination and the number of pigs to be transported. For short distances, the pigs are herded, carried on a bicycle or motorcycle. For long distances to large cities like Gulu, small trucks were used. Traders often came along with their means of transport. There were cases of animal welfare violation reported by traders resulting in death of pigs due to poor handling and conditions during transportation. Instances of pigs being returned to the farmer’s home after failure to find a buyer were reported.

Traders also mentioned that they have some knowledge of checking whether pigs could be infected with the pork tapeworm by examining under the tongue, the eyelids, skin, and cutting the hind leg of a live pig to check for cysts. If the traders find an infected pig at slaughter, it was reported that they returned the carcass to the seller and asked for partial or full refund of the buying price. However, the producers also reported that after refund, the traders sometimes still request to go back with the infected carcass. One of the key informants reported that some producers try to do away with the cysts by scrapping them from the tongue with a knife and reselling the pig after it heals.

*“I am a trader, when I go to buy pig for slaughter, I first examine under the tongues and eyelids for the presence of cysts, and if they are there, I don’t buy that pig,”* Participant, Agago district Mini workshop.

*“They [producers] can also remove the cyst [by scrapping with a knife] under the tongue and they leave the wound to heal for like one or two month and after healing they sale when it actually not been treated,”* District Veterinary Officer KII.

*It also happened to me, they bought my pig and went with it up to Amach, they found my pig that I sold to them had cysts, they carried the pig back to me and they asked me to refund their money but I had already put the money in school fees, I had to struggle to look for this money for paying it back, I gave them a little then I look for the balance and pay later”* P6, female producer FGD, Agago District.

### Slaughtering

Pig slaughter was mainly conducted in undesignated slaughter points (backyard slaughter) with some happening in slaughter points recognized by the district local authority. Most of these locally recognized slaughter points however, lack appropriate infrastructure to enable hygienic and safe procedures to be followed, although meat inspection was sometimes conducted. In the backyard slaughter, each trader or pork joint operator decides where they slaughter from and then carry the carcass to the butchery or pork joint. Backyard slaughters were reported to make it hard for meat inspectors to carry out meat inspection due to inaccessibility, resulting in most pork being sold un-inspected. Traders also reported that sometimes the meat inspectors carry out spot checks on the pork already being sold but noted that they are mostly ill-equipped, lacking means of transport, inspection kits, and personal protective gear.

*“We don’t have a slaughtering point, everyone slaughters from his/her home and take to sell in the center/ pork joint,”* P12, Agago Traders FGD.

*“We don’t have a centralised place where these people should slaughter their pigs you find these pigs are slaughtered in the pork joint and inspectors have to move from one place to another and these places are many so by the time you reach the thirtieth it is almost half sold so half of the pork will have been consumed by consumers of pork,”* District Veterinary Officer KII, Kitgum district.

One meat inspector interviewed approximated that *“one of every four pigs”* that are slaughtered have pork tapeworm cysts in their muscles.

*“I always get like one case porcine cysticercosis in my pork joint every week,” p7, trader FDG, Agago district.*

*“[In] ten pigs, we can get like two or three [that have pork tapeworm cysts], and this is common in the villages where there is poor hygiene and pigs eat faeces anyhow,”* P10, Kitgum traders FGD.

*“A trader from Gulu and bought my pigs, and later found cysts after slaughtering it in Gulu town. The trader brought it back to me, and they told me to look at the condition of the carcass [because it was cystic]. They said we do not want you to incur losses neither should we incur the losses. I returned their money which was Ugx 300,000/=, but they gave me back Ugx 100,000/=, however, they eventually carried back the cystic carcass,”* P5, female producer FGD, Pader district.

Most traders reported that the meat inspectors are few and unable to ensure that all the pork for public sale has been inspected and stamped. It was reported that most meat inspectors even lack technical skills set for pigs and pork inspection, in addition to not appreciating the epidemiology of *T. solium* infections and its potential impacts on human health.

*“There is no place for slaughter and meat inspection is not being done to make pork safe for consumption,”* meat inspector KII, Lamwo district.

Although there are no centralized slaughter points in the four districts, it was reported that some meat inspectors try to inspect the pork at the backyard slaughter points or on the butcheries. However, it was reported that the enforcement of the regulations on condemned meat were weak. This made it possible for pork infected with cysts to be sold to consumers.

*“Sometimes when they [meat inspectors] come across meat infested with cysts they just leave it with butcherman/pork joint operators to hustle with the meat,”* Participant, Agago District Mini workshop.

*“We have to be very open to you; this condemned meat [pork with T. solium cysts] we sell it if the veterinary doctor [meat inspector] doesn’t come to inspect the meat,”* P11, Agago district traders FGD.

### Pork wholesaling

Some traders, after slaughtering the pig, sell the carcasses in wholesale to the small pork joints, bars, roadside roasters, butcheries, and institutions such as schools, restaurants, and community groups. In most cases, it was reported that a full pig carcass of average size cannot be sold completely in 1 day at one butchery/pork joint. The traders therefore slaughter the pig to supply the pork to other pork joints or butchery so that the meat does not spoil.

### Pork retail

Most participants reported that pork retail involved the selling of pork to consumers, normally in piecemeal, such as 1 kg or less. This is carried out by pork joints, butcheries, and roadside roasters. Pork joints are those points where they sell and cook the pork for the consumers. A butchery on the other hand sells only raw pork. Most butcheries are also pork joints and traders reported that it is rare to find a stand-alone butchery or pork joint. Although, some of the buyers knew how to detect cyst-infected pork, it was reported by traders that they sometimes sold pork with cysts if their customers were ignorant or did not care about the presence of cysts. This was more so when the meat inspectors did not inspect the meat, which in most cases was never done. Some consumers were aware of the dangers of consuming pork infected with cysts and checked for the presence of cysts before buying pork.

*“For me before I buy pork from the butcher, I tell the butcher man to first cut some piece of meat and I see so when he cut, I check for the cysts first so when I notice the meat has some cysts I don’t buy,”* respondent, Agago district Mini workshop.

### Consumption

Most participants reported that individual consumers buy ready-to-eat pork for consumption at the pork joint or buy raw pork for family consumption. Most of the participants reported that more men ate pork from pork joints alongside other foods such as cassava, vegetables, and alcohol. Women on the other hand mostly buy and carry raw pork for cooking at home. Pork joint operators noted that wealthier and educated community members rarely buy pork from the local pork joints. Other products bought by consumers included pork oil, and offals.

Consumers raised some common issues that were of food safety concern whenever they set out to consume pork. The most frequently mentioned issues were the presence of cysts in pork, dirty carcasses, poor exsanguination, other disease lesions, uninspected pork, poor hygiene and sanitation of the seller and sales point, lack of sufficient utensils, and poor customer care. In such instances, consumers reported that they would not consider buying from a pork joint or butchery where such concerns are not addressed.

Most consumers reported that the presence of diseases such as ASF and the pork tapeworm were issues of food safety concern, and thus they would only consume pork if it has been inspected by a qualified professional.

“For *me I buy meat which is already inspected because there are some diseases which we cannot detect without the help of the veterinary doctor,”* respondent, Agago district Mini workshop.

Pork tapeworm cysts were reported as a common finding in pork being sold at butcheries and pork joints, and most consumers knew how the cysts look like. The cysts were referred to by local names such as ‘opoto,’ ‘condemned,’ ‘fallen,’ ‘owak,’ among others. In Agago, it was reported that there is a by-law banning sale of pork infested with *T. solium* cysts. However, some consumers said they did not care about buying and consuming pork infected with cysts as they do not think they can harm them, while others understood that proper cooking can kill the cysts.

*“They do not know the effects of cysts on them, I always tell them the effects of cysts on human health, but they will still go ahead and eat. Some of them understand, and they do not eat [pork with cysts],”* Meat Inspector KII, Lamwo district.

For the pork tapeworm control, most stakeholders had a limited understanding of the epidemiology, though they indicated knowing that pork cysts are abnormal and could have adverse health effects. Although, most participants mentioned that they lacked knowledge, some of them were aware of the need to deworm their pigs occasionally or call a veterinarian to treat the pigs.

*“If you get cyst in the pork some people don’t fear the cysts, some will just buy and take them home for consumption even if the meat is contaminated with cyst,”* P4, Lamwo district traders FGD.

### Proposed *Taenia solium* control interventions by the stakeholders

Stakeholders mentioned that the pork tapeworm was a disease they need to control if they are to achieve maximum potential of the pork value chain. The participants from across the four districts identified the following interventions (i) improving access to veterinary services, (ii) posting of public veterinary practitioners in rural areas, (iii) stakeholder sensitization on the control of *T. solium* infections, (iv) increased access to pig drugs and vaccines, (v) establishment of centralized slaughter infrastructures, (vi) building the capacity of meat inspection services, (vii) increase access to quality feed sources, and (viii) integration of animal and human health to address pork tapeworm issues.

*“You know we have pig diseases around, so if the veterinary practitioners would establish drug shops or clinics so that when we have problems with our pigs, we can easily access the services,”* Meat Inspector KII.

*“People in the villages where the veterinary services are inaccessible, eat sick pigs or when they die. But in accessible urban areas [veterinary service providers are nearby], there is strict control [enforcement of meat public health standards],”* Meat Inspector KII, Lamwo district.

*“The government should set an industry for manufacturing of feeds in Agago so as reduce on the expenses of buying feeds from far places like Lamwo, Pader and many more”* respondent, Agago district Mini workshop.

*“For me I think the butcher men should be put in centralised areas where the traders can slaughter their pigs. Because when we slaughter the pigs anyhow the other pigs can also get infection but if we have only one place for slaughtering those cases other diseases will be minimized,”* P4, Lamwo district traders FGD.

## Discussion

This study sought to map the pork value chain in selected districts in Northern Uganda while exploring the factors which help perpetuate the life cycle of *Taenia solium* infections along the pork value chain in northern Uganda and identified opportunities which could be leveraged for control. Using the value chain approach to understand the pork value chain is an approach that has already been used in most East African countries (6, 26, 30). However, this approach has not yet been used to understand the drivers of *T. solium* transmission and the risks along the pork value chain nodes in Uganda. Our findings identify the key actors in the pork value chain and unravel the reasons why *T. solium* remains a key constraint to pig production in Northern Uganda, even when control tools are well known (28).

The pork value chain in Northern Uganda is dominated by disorganized informal low-input, low-output production systems, characterized by a lack of value chain actor institutions, as is characteristic of most low-income countries (30, 33, 34). The largest proportion of the pigs (61–80%) in the region are kept under smallholder extensive systems where the pigs are tethered, partially housed or free ranging most of the time. Low-cost production options such as free roaming and tethering are a preferred production system for the impoverished producers in low-income countries whose key production goal is to meet temporary household needs (34, 35). Even where the producers have pigsties, the pigs are deliberately released to free range during the day and confined at night, to reduce the cost of feeding. Subsistence extensive systems are also widely practiced in other rural areas of Uganda (4, 30). These production methods together with low sanitation levels predispose pigs to health risks including intestinal worms and porcine cysticercosis (17, 36, 37). In this study, most producers understood that roaming pigs were at a considerable risk of disease, but they nevertheless released them, as they believed this made the pigs appear healthier and easier to find boars for mating. The low-input systems are largely practiced since it is light on financial resources, coupled with most farmers perception that pigs can survive on anything. The mindset shift is necessary through extension and other means to enable farmers invest in piggery using appropriate husbandry practices to enable them benefit profitably from piggery without any public health risks. Feeding pigs below their physiological requirement necessitates the need to release them to scavenge in the environment to compensate for the underfeeding. Lack of training, economic constraints, and pig feed shortage were the most mentioned drivers of free ranging production systems, with varying feeding strategies for the wet and the dry seasons.

Variation in preferred feed types during rainy and dry seasons has also been reported in other districts of Uganda (38). Feeding strategies by producers in the study area, whether in the wet or dry season, could expose the pigs to *T. solium* eggs. In the dry season, the pigs are left to roam, eating whatever they find including feces and contaminated shrubs or water. In the wet season, the farmers collect the shrubs and carry them to the pig’s tethering spot or pigsty, potentially also bringing crop residues that are contaminated with feces. Similar practices have been reported in other African countries (34, 39). Thys et al. (34) observed that producers in Zambia were more interested in the perceived benefits of free ranging as they outweighed any associated diseases risks, just as we have observed in this study. Open defecation was widely practiced in the area because of economic and cultural reasons, implying that most environmental surfaces, shrubs, runoff may be contaminated with feces containing *T. solium* eggs.

Interestingly, it was noted that some areas of the study districts, some community members intentionally released pigs to clean the environs of human waste, and the pigs were referred to as ‘sanitation policemen’ or ‘hygiene promoters.’ Possibility of pigs having access to human feces has been reported in many other parts of the world where *T. solium* is endemic (40, 41). The fact that pigs did not seem to be ill made the pork tapeworm a peripheral problem except when incidentally discovered at pig sale or slaughter.

The scarcity of veterinary service providers in the area exacerbates the pig diseases burden along the pork value chain. Veterinary services were important for extension, clinical services, drugs supply, pig movement control, diseases surveillance and control, and pork inspection. The scarcity of veterinarians is not an issue for Northern Uganda alone, but rather a nationwide problem that results in poor pig health, poor welfare observance, low productivity, and profitability of pigs (42, 43). We also found very few women (2/16) among the extension workers, highlighting the gender inequality in extension services. Male domination of the science labor force in Uganda is still a deep-rooted gender inequality, despite the Government’s progressive stand on addressing gender gaps (31, 32). The burden of disease in pigs may be increasing and includes additional human health burdens from zoonotic and foodborne disease (44). As a result, most pork in the region is sold without meat inspection increasing the risk of zoonotic pathogens exposure to the community.

In Uganda, the average extension worker coverage per household ratio is at 1:1800, and the rural areas are at a specific disadvantage as they lack the key basic amenities to attract young professionals. For example, Kitgum district with 39,992 households (45) was served by 7 livestock extension workers (A Kinyera 2022, personal communication, June) translating into one livestock extension worker for 5,708 households, which is much higher than the 1:500 national target (46). As a result, frontline services are offered by unqualified animal health workers who are mostly deficient in key skills requisite for pig diseases prevention and control and have not been registered by the government (47). As a result, pig diseases cannot be effectively controlled, increasing the risk of zoonotic pathogens exposure to the community.

To improve the control of zoonoses, foodborne diseases and AMR, a One Health approach has been recommended. Positively, most districts in Uganda have now formed district one health committees whose role is to champion the management of cross-sectoral public health challenges (48). Unfortunately, though appreciated as endemic, *T. solium* is not a priority zoonosis in Uganda so little attention is given to it by the one health committees and the public health system (49). This could explain why, although the rampant presence of pork infested with cysts in the study area is well acknowledged, there are no deliberate control measures being implemented.

The transportation and aggregation of pigs with minimal or no veterinary oversight also allows for potential spread of pig diseases across the country and beyond. This is exacerbated by porous borders with South Sudan and the Democratic Republic of Congo. The results showed that the traders sometimes transport pigs with cysts to regional markets and returned them to the villages if they went unsold, and without detection by the veterinary inspection system. This shows that diseases including those that are zoonotic can be spread across the country, as with most low-income countries that lack robust and well-regulated public veterinary inspection and surveillance services. Translocating infected pigs and selling pork infested with cysts is not acceptable by public health and animal diseases regulations in Uganda, but they remain unenforceable because of human resource gaps and governance problems (42). Promotion of livestock productivity and trade, often as a pathway out of poverty, with little or no regard for global public health is not just a Ugandan problem but also for most of the developing world (50).

Many actors demonstrated some understanding of the transmission of the pork tapeworm, and many traders knew how the cysts in pork look like and some knew how to check under the tongue for the presence of cysts. However, some consumers of pig were not bothered by the presence of cysts in pork, indicating a level of lack of appreciation of the pork tapeworm lifecycle and the associated effect on their health. This shows the need for a special focus on consumer sensitization on safe pork which would push the entire value chain to supply safe pork. However, the role of public health officials and trained veterinarians is also important. Limited, and fragmented knowledge about *T. solium* transmission has been reported other parts of Uganda (20). Consequently, as was reported by respondents in this study, pork infested with cysts ends up being sold and consumed. There was no clear explanation as to why different actors engage in risky behaviors but lack of knowledge and to some extent ignorance could be the cause, as also found by Ngwili et al. (20).

Lastly, to address the issues that perpetuate *T. solium* endemicity along the pork value chain, actors suggested a couple of interventions that could be implemented to reduce the prevalence of this disease and therefore the health and economic burden on the community. Such interventions would aim to provide a coherent understanding of the *T. solium* infections to actors, support of veterinary public health activities, and improve hygiene and sanitation in the homes. More studies are needed to evaluate the local specific context under which any such interventions will be carried out (25). Sensitization and extension of knowledge to various actors was frequently proposed as one of the key interventions that if implemented could drastically reduce the disease risks to humans and pigs, as most drivers were felt to be tied to ignorance. This has been proposed in other areas across the country (37, 38, 41) but it is not clear if any such findings have been translated into community interventions. Future research within this study area will focus on the co-creation of contextually applicable interventions to protect the community from this important, yet neglected, zoonoses.

## Conclusion

This study highlights a congruence of factors that can aid the transmission of *T. solium* along the pork value chain dominated by informal pig production and marketing. There is a clear need to institute *T. solium* control and prevention interventions engaging with all the actors along the value chain. However, to be better informed about locally appropriate control interventions, it is necessary to first characterize the burden in terms of prevalence of this zoonosis in pigs and humans in the study area using the integrated value chain approaches. We also advocate for improved access to critical pig production inputs and services such as feeds, drugs, extension services, but importantly revamping and realigning the public veterinary services sector to better fulfil its mandate of veterinary extension, animal movement control, pig diseases and surveillance, disease control, and inspection services.

## Data availability statement

The original contributions presented in the study are included in the article/supplementary material, further inquiries can be directed to the corresponding author.

## Ethics statement

The studies involving human participants were reviewed and approved by ILRI Research Ethical committee (Ref. ILRI-IREC2022-09) Vector Control division research ethics committee under the Ministry of Health, Uganda (Ref. VCDREC156). The patients/participants provided their written informed consent to participate in this study.

## Author contributions

NN, SA, SD, LT, and EO: conceptualization and writing—reviewing and editing. NN, SA, LT, and EO: methodology and analysis and data interpretation. NN, SA, and SD: data collection. NN and SA: writing—original draft. All authors contributed to the article and approved the submitted version.

## Funding

This publication was funded by the German Federal Ministry for Economic Cooperation and Development (BMZ) through the One Health Research, Education and Outreach Center in Africa (OHRECA) led by ILRI.

## Conflict of interest

The authors declare that the research was conducted in the absence of any commercial or financial relationships that could be construed as a potential conflict of interest.

## Publisher’s note

All claims expressed in this article are solely those of the authors and do not necessarily represent those of their affiliated organizations, or those of the publisher, the editors and the reviewers. Any product that may be evaluated in this article, or claim that may be made by its manufacturer, is not guaranteed or endorsed by the publisher.

## Footnotes

^1^https://www.qsrinternational.com/nvivo-qualitative-data-analysis-software/home

^2^https://cmap.ihmc.us/
